# Implementation and Validation of an Analytical Method for Lincomycin Determination in Feathers and Edible Tissues of Broiler Chickens by Liquid Chromatography Tandem Mass Spectrometry

**DOI:** 10.1155/2019/4569707

**Published:** 2019-02-25

**Authors:** Aldo Maddaleno, Ekaterina Pokrant, Francisca Yanten, Betty San Martin, Javiera Cornejo

**Affiliations:** ^1^Laboratory of Veterinary Pharmacology, Faculty of Veterinary and Animal Sciences, University of Chile, 8820808 Santiago, Chile; ^2^Food Safety Unit, Preventive Medicine Department, Faculty of Veterinary and Animal Sciences, University of Chile, 8820808 Santiago, Chile

## Abstract

Recent studies have detected different antimicrobial residues in broiler chicken feathers, where they persisted for longer periods of time and at greater concentrations than in edible tissues. However, until today, lincomycin behaviour in this nonedible tissue has not been assessed yet. Considering this, an analytical methodology to detect and quantify this antibiotic concentration in feathers, muscle, and liver tissues from broiler chickens was implemented and in-house validated. The methodology will allow the determination of the bioaccumulation of this highly persistent antibiotic in feathers of treated birds. For this purpose, 98% lincomycin and 95% lincomycin D3 standards were used. Methanol was selected as the extraction solvent, and Chromabond® Florisil® cartridges were used for the clean-up stage. The separation of analytes was performed through the analytical column SunFire C18 with a running time of 4 minutes, and the instrumental analysis was performed through an LC-MS/MS, with a liquid chromatograph Agilent® 1290 Infinity, coupled to an AB SCIEX® API 5500 mass spectrometer. An internal protocol for an in-house validation was designed based on recommendations from Commission Decision 2002/657/EC and the Guidance document on the estimation of limit of detection and limit of quantification for measurements in the field of contaminants in feed and food. The average retention time for lincomycin was 2.255 min (for quantifier ion, 126.0). The calibration curves showed a coefficient of determination (*r*^2^) greater than 0.99 for all matrices, while recovery levels ranged between 98% and 101%. The limit of detection (LOD) calculated was of 19, 22, and 10 *μ*g·kg^−1^, and the limit of quantification (LOQ) was of 62, 73, and 34 *μ*g·kg^−1^ in feathers, muscle, and liver, respectively. This method detects lincomycin in the studied matrices, confidently and accurately, as it is required for designing analytical studies of drug residues in edible and nonedible tissues, such as feathers.

## 1. Introduction

Antimicrobials have been used therapeutically in diverse areas of animal farming for the treatment of different bacterial pathologies. Treating these animals not only controls pathogens affecting their own health, but it also helps to control human diseases. Additionally, antimicrobials have long been used with the intention of leveraging the efficiency of animal production [1].

Lincomycin is a natural antimicrobial belonging to the lincosamides class, and it is synthesised by *Streptomyces lincolnensis* bacteria. This drug is recommended for the treatment of diseases caused by aerobic and anaerobic Gram-positive infections, such as *Staphylococcus* spp. and *Streptococcus* spp. It is also used in association with other antimicrobial drugs to treat livestock infections caused by *Bacteroides fragilis*, as well as diseases of the respiratory tract in different animal species. The main mechanism of action of lincosamides focus on blocking protein synthesis in bacteria. It affects several activation steps of amino acid monomers via the aminoacyl-tRNA, as well as the processes of initiation, elongation, and termination of the polypeptide chains at the level of the bacterial ribosome [2].

Lincosamides and macrolides are first-choice antimicrobials that are used as bacteriostatic drugs in veterinary microbiology and as bactericidal drugs in farm animals [3]. However, using these antimicrobials in humans presents some risks due to their ability to cross different barriers, either in individuals or the environment, thereby helping to increase the selection of resistant bacteria at a global scale. Different surveillance studies have shown that foods of animal origin may present residues of these drugs, after it was administered to farm animals in several geographical areas [4, 5]. These residues have even been found in both environment and in animals that were not treated with lincomycin themselves [6]. This finding proves that these residues are transferred from the production chain to the environment via organic waste, such as urine and faeces from treated animals or from other elements not yet qualified or quantified [6].

Several studies that have developed and validated methods according to EU Commission Decision 2002/657/EC have pointed out that antibiotics can be detected in several edible tissues and other products like honey [7–9]. Specifically, lincomycin residues can be found in different animal products and tissues, such as muscle and plasma [2, 10–12]. From this evidence, it is possible to infer that other animal structures, as feathers, could accumulate antimicrobial residues as well. In this regard, recent studies have used chromatographic methodologies to detect drug residues of veterinary importance. In those studies, researchers found that residues of enrofloxacin, flumequine, oxytetracycline, florfenicol, and tylosin were transferred to feathers of treated broiler chicken [13–17]. Furthermore, these residues were found for longer periods, and at higher concentrations, than those detected in edible tissues. Current regulations from the European Commission, aimed at controlling these drug residues in foods of animal origin, have set a maximum residue level (MRL) for lincomycin in any farm animal species: 100 *μ*g·kg^−1^ for muscle tissue, 50 *μ*g·kg^−1^ for fat tissue and eggs, 500 *μ*g·kg^−1^ for liver tissue, and 1500 *μ*g·kg^−1^ for kidney tissue, whereas for cattle milk, the MRL is 150 *μ*g·kg^−1^ [18]. Meanwhile, regulations from the Codex Alimentarius set a limit of 200 *μ*g·kg^−1^ for muscle tissue, 500 *μ*g·kg^−1^ for liver tissue, and 100 *μ*g·kg^−1^ for fat tissue, as well as 500 *μ*g·kg^−1^ and 1500 *μ*g·kg^−1^ for kidney tissue of poultry and swine, respectively [19]. Neither regulation includes poultry feathers, as these structures are not meant for direct consumption. However, residues from this antimicrobial could actually be present in them. Importantly, these by-products are currently being processed to prepare feather meal, which is used as an ingredient for animal diet formulations. Thus, it must be considered the possibility that these residues could be present in diets that include this ingredient as a food additive [20]. Bearing in mind the consequences that this residue may unleash if it is reintroduced in stage of the production chain, it becomes necessary to develop quantitative methodologies for analysis of lincomycin in feathers. A quantitative and confirmatory LC-MS/MS method will allow to avoid the re-entrance of these antibiotic residues in the food chain. Several researchers have implemented methodologies on the basis of microbiological systems of chemiluminescent electromigration in different matrices; however, their results have not been optimum in terms of sensitivity and stability [21]. Other methodologies that have been attempted were based on immunochromatographic assays and are intended for the determination of lincomycin in milk, honey, muscle tissues, and urine [22]. In the case of LC-MS/MS, other researchers have tried using this kind of methodologies for the analysis of animal products, such as muscle tissue, honey, milk, and eggs [23–25]. For example, in a study reported by Jansen et al., the authors describe a qualitative methodology for the determination of several antimicrobials, including the lincomycin class [26]. However, it is important to also determine quantitatively the residues that could be transferred to feathers and other important matrices, such as liver and muscle tissue from birds that have been treated with this drug. Those results would allow that the behaviour of lincomycin could be determined in those by-products. Despite previous studies, they have determined the concentration and depletion time of different antibiotics in feathers [13–17]. Currently, the behaviour of lincomycin in this matrix has not been studied.

Lincomycin residues in feathers can become a re-entry path for these drug residues into the food chain, if they are used in the formulation of animal diets. The demonstrated persistence of different drug residues in feathers poses a risk to public health due to the probability of becoming an unknown route of antibiotic cross contamination. Love et al. described the risk related to the administration of contaminated feather meal in diets of food animals. In this study, of the 46 antimicrobials that were tested, over one-third (*n*=17) were detected in feather meal samples. This information provides a clear overview about the trespassing of residues into feathers and the possibility to become a risk in the food chain [27]. Therefore, the implementation of an analytical method in feathers is critical to properly assess the bioaccumulation of this drug in this matrix.

In this work, we have implemented an optimized LC-MS/MS analytical methodology. The method was validated according to an internal protocol based on Decision 2002/657/EC and the Guidance document on the estimation of limit of detection and limit of quantification for measurements in the field of contaminants in feed and food [28, 29]. This method allowed to accurately and confidently quantify lincomycin residues in feathers and edible tissues from broiler chickens. This LC-MS/MS analytical methodology allows the quantification and confirmation of lincomycin in broiler chicken feathers, muscle, and liver.

## 2. Materials and Methods

### 2.1. Standard Solutions

A lincomycin standard of 98% certified purity—manufactured by Dr. Ehrenstorfer, GmbH (Augsburg, Germany)—and a lincomycin D3 standard of 95% certified purity—manufactured by Toronto Research Chemicals (Toronto, Canada)—were used to prepare stock solutions by dissolving 1000 *µ*g·mL^−1^ of these compounds in a methanol/water (50 : 50) solution.

Working solutions were then prepared from the stock solutions by diluting 2000 ng·mL^−1^ and 1000 ng·mL^−1^ of lincomycin and lincomycin D3 solutions, respectively. All of these solutions were individually stored in microcentrifuge tubes at −80°C.

### 2.2. Chemicals and Reagent

Before the extraction stage, water was distilled and deionised in the laboratory using the Milli-Q® system with a resistance of less than 18.2 MΩ (Merck KGaA, Burlington, Massachusetts, USA).

This method also required reagents such as water, methanol, and acetonitrile. These reagents were of HPLC-grade purity and manufactured by J.T.Baker® (Avantor® Performance Materials LLC, Center Valley, PA) or a similar brand. Other reagents such as n-hexane, ethyl acetate, and acetic acid were of HPLC-grade purity and sourced from the line of LiChrosolv® solvents (Merck KGaA, Darmstadt, Germany). Meanwhile, ammonium acetate was of P.A. grade purity and sourced from the line of LiChrosolv® solvents (Merck KGaA, Darmstadt, Germany).

Solid-phase extraction cartridges were selected from the Chromabond® Florisil® line and manufactured by Macherey-Nagel GmbH and Co. KG (Düren, Germany).

### 2.3. Instrumentation

All samples were analysed using a liquid chromatograph device from the Agilent® 1290 Infinity Series coupled to an AB Sciex® API 5500 triple quadrupole mass spectrometer (AB Sciex LLC, Framingham, MA), which was fitted with a SunFire® C18 analytical column of 3.5 *μ*m 2.1 × 150 mm manufactured by Waters® (Waters Corporation, Milford, MA). The chromatographic separation procedure involved a mobile phase at pH 3.5 ± 0.2 made from two solvents (65% solvent A, 35% solvent B). Solvent A was a solution of 0.02% ammonium acetate at pH 4.5 ± 0.05, whereas solvent B was a solution of 0.1% acetic acid in acetonitrile. The gradient flow was set at 200 *μ*l·min^−1^, the gradient elution was from 0 to 4 minutes (65% solvent A, 35% solvent B), the injection volume was 5 *μ*L, and the column oven temperature was of 30°C.  Table 1 lists the parameters used for the operation of the mass detector.  Table 2 lists the ion masses that were monitored in this study.

Lastly, the equipment was managed and integrated using the Analyst® 1.6.3 (AB Sciex LLC, Framingham, MA) and MultiQuant® 3.0 (AB Sciex LLC, Framingham, MA) software packages, respectively.

### 2.4. Sample Processing

Samples were sourced from commercial broiler chickens and were first analysed by HPLC-MS/MS to confirm the absence of lincomycin residues. Feather samples were cryogenically treated with liquid nitrogen to ease their grinding in a Robot Coupe® R4 “table-top cutter” food processor (Robot Coupe®, Vincennes, France). Likewise, muscle and liver tissue samples were also ground in the food processor, though no liquid nitrogen processing was required for those samples.

### 2.5. Extraction Procedure for Feathers, Muscle, and Liver Samples

The extraction of lincomycin residues from feather samples began by weighing in 1.00 ± 0.01 g of each sample in a 50 mL polypropylene tube. These samples were then fortified with the lincomycin standard, as well as the lincomycin D3 internal standard. Subsequently, 40 mL of HPLC-grade methanol were added to the sample tubes before these were shaken in a Multi Reax® agitator (Heidolph Instruments GmbH and Co. KG, Schwabach, Germany). Afterwards, tubes were sonicated and centrifuged in a Hettich® ROTOFIX 32A centrifuge (Hettich Lab Technology, Beverly, Massachusetts) at 2,700 g for 15 minutes for feathers, 10 minutes for muscle, and 15 minutes for liver samples. The resulting supernatant was filtered through glass fibre and then passed at a flow rate of 1 mL·min^−1^ through a Chromabond® Florisil® cartridge (Macherey-Nagel GmbH and Co. KG, Düren, Germany). This cartridge was previously conditioned with 10 mL of HPLC-grade hexane and 10 mL of a solution (8 : 2) of HPLC-grade methanol and HPLC-grade ethyl acetate. This filtrate was collected in a 50 mL falcon tube and then evaporated, under a mild nitrogen flow, in a water bath set at a temperature of 40–50°C. Samples were reconstituted in 500 *μ*L of a solution (9 : 1) of methanol and HPLC-grade water. Once reconstituted, samples were shaken, sonicated, and centrifuged in a VWR® 2417R (Avantor, Radnor, PA) device at 17,136 g for 5 minutes for feathers, 5 minutes for muscle, and 10 minutes for liver samples. Finally, samples were transferred to a glass vial using a Millex® (Merck KGaA, Burlington, Massachusetts, USA) 33 mm polyvinylidene fluoride (PVDF) sterile filter syringe.

The extraction procedure for muscle and liver samples followed the same principles than the protocol designed for feather samples, with the sole exceptions of using 20 mL of solvent for the methanol extraction step and that samples were not prefiltered through glass wool before passing through the Chromabond® Florisil® cartridges (Macherey-Nagel GmbH and Co. KG, Düren, Germany).

### 2.6. Validation Procedure

To complete the in-house validation of these analytical methods was followed an internal protocol specially designed for this study based on recommendations from the European Commission Decision 2002/657/EC and the Guidance document on the estimation of limit of detection and limit of quantification for measurements in the field of contaminants in feed and food [28, 29]. Due to the impact that could have contaminations with pharmaceutical residues in products used as additives in the feeding of animals destined for consumption, it was decided that these guides offered the minimum analytical base to establish the statistical parameters of the methodology and to accomplish with the current regulations focused on this type of analysis.

This single validation assessed parameters such as recovery performance, precision (measured as repeatability and intralaboratory reproducibility), linearity, retention time, limit of detection (LOD), and limit of quantification (LOQ).

To assess recovery performance, all samples of feather, muscle, and liver tissues were analysed to certify them as blank, ruling out any contamination with lincomycin residues. Then, samples were fortified at 0.2, 0.8, and 1.6 times the MRL, which has been set at 100 *μ*g·kg^−1^ by European Commission for muscle tissue (37/2010/EC) [18]. With this value selection of 20 *μ*g·kg^−1^, all the types of samples were analysed with a detection limit under the MRL set for muscle by the European Commission and in a level that allows to reliably detect low concentrations of the residue in feathers. The recovery performance of the extraction stage for each level was calculated by comparing samples results against those from injections of pure standard solutions at the same concentration level.

Precision was assessed by its components: repeatability and intralaboratory reproducibility. In the case of repeatability, six sample sets were fortified and processed at three different concentration levels (20, 80, and 160 *µ*g·kg^−1^), on the same day. Meanwhile, intralaboratory reproducibility was measured by using six sample sets, fortified at the same concentration levels than for the repeatability assessment, but these were analysed on different days and by different analysts.

As for the linearity of these methods, it was assessed by plotting calibration curves for each matrix, at five different concentration levels (20, 40, 80, 120, and 160 *µ*g·kg^−1^).

The selectivity and specificity of these methods were assessed by analysing blank samples of feather, muscle, and liver tissues from different sources. LOD and LOQ were determined on the basis of 10 spiked blank samples of each matrix. The criteria for selecting the LOD of this method was to achieve a signal-to-noise ratio greater than 3 : 1, whereas the LOQ is the concentration that gives a signal-to-noise ratio greater than 10 : 1.

## 3. Results and Discussion

### 3.1. Method Development and Optimization

As mentioned before, our research group developed a method for detection of lincomycin residues in feathers, muscle, and liver tissues and in-house validated it based on the recommendations described in the Commission Decision 2002/657/EC and Guidance document on the estimation of limit of detection and limit of quantification for measurements in the field of contaminants in feed and food [28, 29].

A quick, easy, and inexpensive extraction methodology enhances the capability of every laboratory to implement any analytical method that might be technically qualified to perform. The method implemented in this work allows for the simultaneous analysis of three different matrices to detect lincomycin residues, and it does not require modifications of any of the chromatographic conditions of the mass spectrometer. Is important to emphasise that the SunFire® C18 chromatographic column was selected for this particular method due to its resolution, being able to distinguish among interfering residues with similar molecular weights and chemical characteristics. Such versatility enhanced the specificity of the analytical method.

The solvents that were used for the extraction stage are the base for several methodologies. Using such solvents for the implementation of this method greatly facilitates its adoption in most analytical laboratories that currently work with chromatographic techniques. The fact that this method provides robust results in these three matrices, with only a few modifications in methanol extraction volume and glass fibre prefiltration, also favours its suitability for simultaneously analysing all these matrices.

The method detected residues using chromatographic techniques, monitoring their masses and specific retention times.  Table 3 specifically lists average retention times and coefficients of variation for six analyses of certified standards of lincomycin and lincomycin D3. Fragment ion 407.0/126.0 was used to quantify lincomycin in all three matrices, whereas ion 407.0/359.0 was confirmatory due to its chromatographic intensity.

### 3.2. Selectivity and Specificity

**Figure 1:**
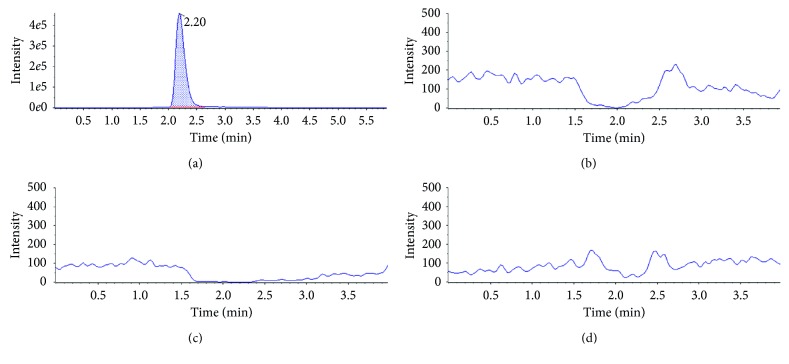
Chromatograms of lincomycin from (a) a pure standard solutions injection, (b) a blank feather sample, (c) a blank muscle sample, and (d) a blank liver sample.

No interfering signals were detected in any of the three matrices, around the retention time that is characteristic of lincomycin residues, for the sample group comprising 20 blank samples from different sources (Figure 1). Therefore, this parameter met the acceptance criteria, being the specific method for the three study matrices.

### 3.3. Detection Range

**Figure 2:**
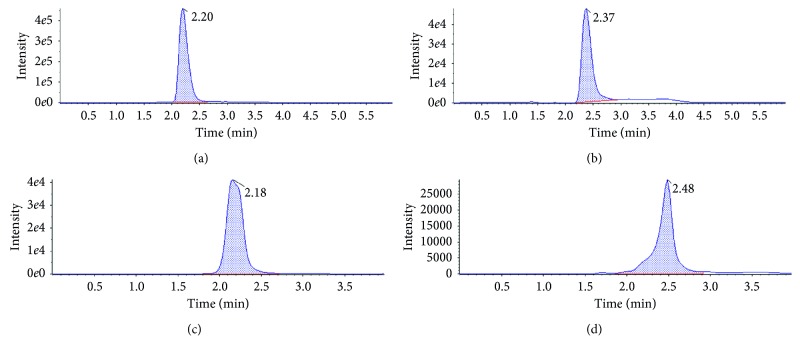
Chromatograms of lincomycin (a) from lincomycin pure standard injection and (b) feather, (c) muscle, and (d) liver samples fortified with lincomycin standard at a concentration of 20 *µ*g·kg^−1^.

The LOD was set at 19, 22, and 10 *µ*g·kg^−1^ for feather, muscle, and liver, with a signal-to-noise ratio greater than 3 : 1 for the three matrices. Our results indicated that for 10 repetitions (fortified at 20 *µ*g·kg^−1^) in all matrices (Figure 2), the relative standard deviation of the replicates was less than 10%, which accomplished the acceptance criteria for the parameter.

The LOQ was defined as 3.3 times the LOD previously calculated with the deviation standard of the 10 repetitions. These values for LOQ were accepted because they reached a signal-to-noise ratios greater than 10 : 1 for the analyte in all matrices.  Table 4 lists the LOD, average concentrations detected from these 10 samples (fortified at 20 *µ*g·kg^−1^), as well as their respective standard deviations, relative standard deviation (RSD), and LOQ for the three matrices.

### 3.4. Calibration Curves

Each calibration curve comprised five concentration levels: 20, 40, 80, 120, and 160 *µ*g·kg^−1^. These concentration points in the calibration curve were determined to explore the lincomycin levels in feathers because there is no previous information regarding concentrations in this matrix or a reference value to follow Thus, the selection of 20 *μ*g·kg^−1^ as a first point of the calibration curve to determinate the levels of the residue in feathers was used like an explorative value and no has relation with the actual MRL in muscle and liver.

The slope is the most useful parameter for the analysis of linear equations, as it provides the best information about sensitivity of analytical methods and their quantification capabilities. Thus, we calculated these slopes, as well as their coefficient of variation. Our results showed that the coefficients of determination (*r*^2^) for the calibration curves were higher than 0.99, and their coefficients of variation were lower than 25%. Therefore, the acceptance criteria were met, and a high sensitivity was achieved in the three study matrices.  Table 5 lists the average of coefficients of determination, slope average, and their respective coefficient of variation, for all matrices.

### 3.5. Recovery and Precision

Recovery percentages for all matrices averaged between 98.47% and 100.67%. The results obtained meet the acceptance criteria since they fall within the range indicated by the Commission Decision 2002/65/EC, which corresponds to values between 80% and 110%. Therefore, this method has a reliability sufﬁcient to quantify lincomycin in feathers, muscle, and liver.

In the case of the precision, the obtained results for repeatability did not exceed the values of intralaboratory reproducibility, and this did not exceed an RSD of 23%.  Table 6 shows average recoveries as well as their respective RSD for these three matrices, according to their working concentration. In addition, the results of the precision through repeatability and intralaboratory reproducibility are shown.

Therefore, all parameters accomplished the acceptance criteria set by the European Commission 2002/657/EC and the Guidance document on the estimation of limit of detection and limit of quantification for measurements in the field of contaminants in feed and food for the detection of lincomycin in these matrices accurately and reliably.

## 4. Conclusions

In the present study, a confirmatory analytical method for detecting lincomycin in feathers, muscle, and liver samples was developed and in-house validated. This analytical method is reliable and capable of determining residue concentrations in these matrices. Furthermore, they exhibit results meet the criteria set forth by the Commission Decision 2002/657/EC and the Guidance document on the estimation of limit of detection and limit of quantification for measurements in the field of contaminants in feed and food. Therefore, this work could become the basis for future research on the behaviour of lincomycin in feathers sourced from birds that have received therapeutic doses of this drug. The confirmatory nature of this LC-MS/MS analytical method makes it a reliable tool for developing further studies to determine the behaviour, transfer, and depletion of lincomycin in feathers, muscle, and liver from broiler chickens. This study is essential for the development of control measures and surveillance strategies that assess lincomycin residues in this by-product.

## Figures and Tables

**Table 1:** Operation parameters of the MS/MS detector.

Parameter	Analytical conditions
Ionisation	ESI
Scan type	MRM
TEM	500°C
GS1	60 psi
GS2	40 psi
CUR	25 psi
CAD	8 psi
IS	3600 V
Total scan time	1.62 sec

ESI: electrospray ionisation; MRM: multiple reaction monitoring; TEM: source temperature; GS1: nebuliser; GS2: turbo ion; CUR: curtain gas; CAD: collision gas; ISV: ion spray voltage.

**Table 2:** Monitored ion masses.

Analyte	Precursor ion (Q1 mass) (Da)	Fragment ion (Q3 mass) (Da)	Time (ms)	DP (V)	EP (V)	CE (V)	CXP (V)
Lincomycin 1	407.0	126.0	400.0	26.0	6.0	30.0	12.0
Lincomycin 2	407.0	359.0	400.0	26.0	6.0	27.0	4.0
Lincomycin D3 1	410.0	129.0	400.0	26.0	6.0	30.0	12.0
Lincomycin D3 2	410.0	362.0	400.0	26.0	6.0	27.0	12.0

Q1: quadrupole 1; Q3: quadrupole 3; Da: dalton; DP: declustering potential; EP: entrance potential; CE: collision energy; CXP: collision cell exit potential; V: volt. The precursor ion 410.0/362.0 of lincomycin D3 was used for quantification of the analyte in all samples.

**Table 3:** Average retention time and relative standard deviation (RSD) of monitored ion masses of lincomycin and lincomycin D3 residues.

Analyte	Precursor ion (Da)	Fragment ion (Da)	Average RT (min)	RSD (%)
Lincomycin	407.0	126.0^*∗*^	2.255	0.24
		359.0^*∗∗*^	2.247	0.36

Lincomycin D3	410.0	362.0	2.237	0.23

Da: dalton; RT: retention time; RSD: relative standard deviation; ^*∗*^quantifier ion; ^*∗∗*^confirmatory ion.

**Table 4:** Limit of detection (LOD), average concentration (calculated from 20 samples fortified up to the LOD concentration), standard deviation (SD), relative standard deviation (RSD), and limit of quantification (LOQ) for lincomycin in samples of feather, muscle, and liver tissues.

Biological matrix	LOD (*µ*g·kg^−1^)	Average concentration (*µ*g·kg^−1^)	SD	RSD (%)	LOQ (*µ*g·kg^−1^)
Feather	19	20.80	1.52	7.29	62
Muscle	22	21.09	0.17	0.78	73
Liver	10	21.80	2.17	9.96	34

**Table 5:** Method linearity parameters for three calibration curves: *r*^2^ average, slope average, and their respective relative standard deviation (RSD) for lincomycin, by biological matrix.

Biological matrix	*r* ^2^ average	RSD (%)	Slope average	RSD (%)
Feather	0.998	0.13	0.241	1.63
Muscle	0.999	0.01	0.029	1.44
Liver	0.995	0.15	0.650	4.33

**Table 6:** Precision parameters: repeatability, intralaboratory reproducibility, and average recovery rate with their respective relative standard deviation (RSD), for each biological matrix, at concentrations of 20, 80, and 160 *µ*g·kg^−1^.

Biological matrix	Working concentration (*µ*g·kg^−1^)	Repeatability RSD (%)	Intralaboratory reproducibility RSD (%)	Average recovery	Recovery RSD (%)
Feathers	20	6.6	9.1	100.02	0.06
	80	2.9	4.0	99.99	0.03
	160	0.6	0.8	100.00	0.01

Muscle	20	6.9	11.2	98.78	0.04
	80	3.0	4.8	100.53	0.02
	160	0.6	1.0	99.89	0.004

Liver	20	12.5	20.7	98.47	0.09
	80	5.1	8.5	100.67	0.04
	160	1.1	1.9	99.86	0.01

## Data Availability

The data used to support the findings of this study are available from the corresponding author upon request.
